# Bis(chloro­acetato-κ^2^ *O*,*O*′)bis­(2-fluoro­benzyl-κ*C* ^1^)tin(IV)

**DOI:** 10.1107/S1600536811051002

**Published:** 2011-11-30

**Authors:** Aixia Deng, Mouyong Teng, Qian Xie, Nana Yan

**Affiliations:** a College of Materials Science and Engineering, Liaocheng University, Shandong 252059, People’s Republic of China

## Abstract

In the title complex, [Sn(C_2_H_2_ClO_2_)_2_(C_7_H_6_F)_2_], the Sn^IV^ atom is located on a twofold rotation axis and forms a strongly distorted *trans*-octa­hedral geometry. The equatorial plane is defined by two chelating chloro­acetate ligands with asymmetrical Sn—O bond lengths, while the axial positions are occupied by the C atoms of two 2-fluoro­benzyl groups. In the crystal, infinite chains in the [010] direction are formed through inter­molecular Sn⋯O inter­actions [Sn⋯O separation = 3.682 (3) Å].

## Related literature

For details of the synthesis, see: Zhang *et al.* (2007 ▶).
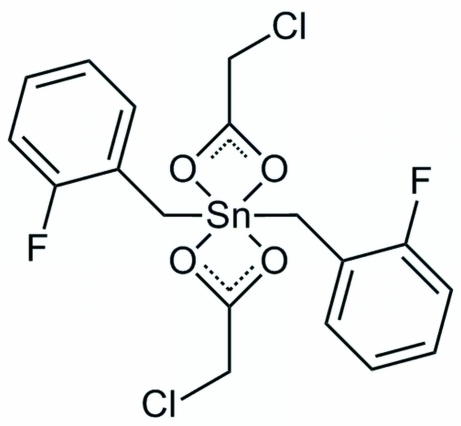


## Experimental

### 

#### Crystal data

[Sn(C_2_H_2_ClO_2_)_2_(C_7_H_6_F)_2_]
*M* *_r_* = 523.90
Monoclinic, 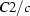

*a* = 17.3841 (18) Å
*b* = 5.0480 (8) Å
*c* = 22.808 (2) Å
β = 93.760 (1)°
*V* = 1997.2 (4) Å^3^
*Z* = 4
Mo *K*α radiation
μ = 1.59 mm^−1^
*T* = 298 K
0.29 × 0.15 × 0.12 mm

#### Data collection

Bruker SMART 1000 CCD area-detector diffractometer
Absorption correction: multi-scan (*SADABS*; Bruker, 2001 ▶) *T*_min_ = 0.656, *T*_max_ = 0.833
4738 measured reflections
1754 independent reflections
1551 reflections with *I* > 2σ(*I*)
*R*_int_ = 0.032

#### Refinement

*R*[*F* ^2^ > 2σ(*F* ^2^)] = 0.033
*wR*(*F* ^2^) = 0.071
*S* = 1.00
1754 reflections
123 parameters
H-atom parameters constrained
Δρ_max_ = 0.71 e Å^−3^
Δρ_min_ = −0.26 e Å^−3^

### 

Data collection: *SMART* (Bruker, 2007 ▶); cell refinement: *SAINT* (Bruker, 2007 ▶); data reduction: *SAINT*; program(s) used to solve structure: *SHELXS97* (Sheldrick, 2008 ▶); program(s) used to refine structure: *SHELXL97* (Sheldrick, 2008 ▶); molecular graphics: *SHELXTL* (Sheldrick, 2008 ▶); software used to prepare material for publication: *SHELXTL*.

## Supplementary Material

Crystal structure: contains datablock(s) I, global. DOI: 10.1107/S1600536811051002/bh2395sup1.cif

Structure factors: contains datablock(s) I. DOI: 10.1107/S1600536811051002/bh2395Isup2.hkl

Additional supplementary materials:  crystallographic information; 3D view; checkCIF report

## Figures and Tables

**Table 1:** Selected bond lengths (Å)

Sn1—O1	2.109 (2)
Sn1—C3	2.121 (4)
Sn1—O2	2.537 (3)

**Fig. 1.**
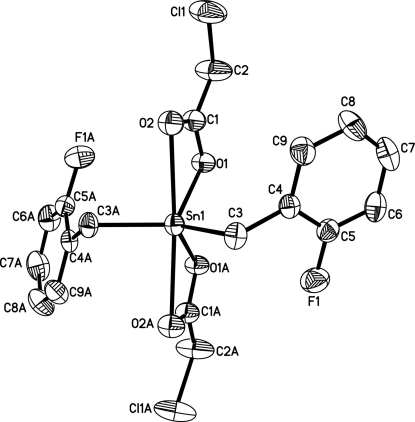
The molecular structure of the title compound, showing 30% probability displacement ellipsoids.

**Fig. 2.**
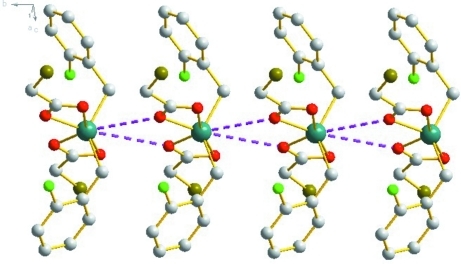
The unit cell of the title compound.

## supplementary crystallographic information

### Comment

The title complex was obtained using a route similar to that used for the synthesis of a trinuclear tin complex (Zhang *et al.*, 2007). The title complex has 2-fold symmetry, with the Sn atom placed on the crystallographic symmetry axis (Fig. 1). Selected bond lengths and angles are given in table 1. The coordination geometry of tin can be described as a distorted *trans*-octahedron geometry, with two C atoms of 2-fluorobenzyl groups occupying the axial positions. The C3—Sn1—C3*^i^* bond angle of 133.6 (2)° (symmetry code *i*: 1 - *x*, *y*, 1/2 - *z*) reflects the distortion from octahedral geometry. The equatorial plane is defined by four O atoms of two symmetry-related chloroacetate ligands. The bond lengths in the equatorial plane are Sn1—O1 = 2.109 (2) and Sn1—O2 = 2.537 (3) Å, reflecting the asymmetrical coordination of acetate groups.

The complex forms infinite chains containing Sn_2_O_2_ rings, through intermolecular Sn···O contacts, characterized by separations Sn···O = 3.682 (3) Å (Fig. 2).

### Experimental

Chloroacetic acid (2 mmol) was added to a sodium ethoxide solution (2 mmol, 20 ml of ethanol), and the mixture was stirred for 30 min. Then, 1 mmol of bis(2-fluorobenzyl)tin(IV)dichloride (Zhang *et al.*, 2007) was added to the mixture, continuing the reaction for 12 h at 318 K. After cooling down to room temperature, the reaction was filtered off. The solvent of the filtrate was gradually removed by evaporation under vacuum, until a solid product was obtained. The solid was recrystallized from ether-dichloromethane and colourless crystals suitable for X-ray diffraction were obtained (m.p. 464 K). Analysis calculated for C_18_H_16_Cl_2_F_2_O_4_Sn: C 41.26, H 3.08%; found: C 41.29, H 3.06%.

### Refinement

All H atoms were placed geometrically and treated as riding on their parent atoms with C—H bond lengths fixed to 0.93 Å for aromatic CH and 0.97 Å for methylene CH_2_. Isotropic displacement parameters for H atoms were calculated as *U*_iso_(H) = 1.2*U*_eq_(carrier C).

### Crystal data

**Table:**

[Sn(C_2_H_2_ClO_2_)_2_(C_7_H_6_F)_2_]	*F*(000) = 1032
*M**_r_* = 523.90	*D*_x_ = 1.742 Mg m^−^^3^
Monoclinic, *C*2/*c*	Melting point: 464 K
Hall symbol: -C 2yc	Mo *K*α radiation, λ = 0.71073 Å
*a* = 17.3841 (18) Å	Cell parameters from 1705 reflections
*b* = 5.0480 (8) Å	θ = 2.9–25.0°
*c* = 22.808 (2) Å	µ = 1.59 mm^−^^1^
β = 93.760 (1)°	*T* = 298 K
*V* = 1997.2 (4) Å^3^	Block, colourless
*Z* = 4	0.29 × 0.15 × 0.12 mm

### Data collection

**Table:**

Bruker SMART 1000 CCD area-detector diffractometer	1754 independent reflections
Radiation source: fine-focus sealed tube	1551 reflections with *I* > 2σ(*I*)
graphite	*R*_int_ = 0.032
φ and ω scans	θ_max_ = 25.0°, θ_min_ = 1.8°
Absorption correction: multi-scan (*SADABS*; Bruker, 2001)	*h* = −20→17
*T*_min_ = 0.656, *T*_max_ = 0.833	*k* = −5→6
4738 measured reflections	*l* = −27→22

### Refinement

**Table:**

Refinement on *F*^2^	Primary atom site location: structure-invariant direct methods
Least-squares matrix: full	Secondary atom site location: difference Fourier map
*R*[*F*^2^ > 2σ(*F*^2^)] = 0.033	Hydrogen site location: inferred from neighbouring sites
*wR*(*F*^2^) = 0.071	H-atom parameters constrained
*S* = 1.00	*w* = 1/[σ^2^(*F*_o_^2^) + (0.0342*P*)^2^] where *P* = (*F*_o_^2^ + 2*F*_c_^2^)/3
1754 reflections	(Δ/σ)_max_ < 0.001
123 parameters	Δρ_max_ = 0.71 e Å^−^^3^
0 restraints	Δρ_min_ = −0.26 e Å^−^^3^
0 constraints	

### Fractional atomic coordinates and isotropic or equivalent isotropic displacement parameters (Å^2^)

**Table:**

	*x*	*y*	*z*	*U*_iso_*/*U*_eq_	
Sn1	0.5000	0.07163 (7)	0.2500	0.04380 (15)	
Cl1	0.58874 (11)	0.2741 (3)	0.03745 (5)	0.1169 (6)	
F1	0.29621 (15)	0.3089 (6)	0.24062 (11)	0.0831 (8)	
O1	0.52467 (15)	0.3929 (5)	0.19515 (10)	0.0517 (7)	
O2	0.55526 (17)	0.0310 (5)	0.15017 (12)	0.0602 (7)	
C1	0.5493 (2)	0.2692 (9)	0.15046 (17)	0.0542 (10)	
C2	0.5669 (4)	0.4449 (10)	0.0999 (2)	0.098 (2)	
H2A	0.6101	0.5583	0.1120	0.117*	
H2B	0.5227	0.5579	0.0903	0.117*	
C3	0.3963 (2)	−0.0939 (7)	0.21189 (17)	0.0522 (10)	
H3A	0.4080	−0.2616	0.1937	0.063*	
H3B	0.3620	−0.1296	0.2428	0.063*	
C4	0.3557 (2)	0.0799 (8)	0.16691 (16)	0.0465 (9)	
C5	0.3052 (2)	0.2743 (9)	0.18211 (18)	0.0537 (10)	
C6	0.2662 (3)	0.4379 (9)	0.1434 (2)	0.0703 (13)	
H6	0.2318	0.5635	0.1561	0.084*	
C7	0.2789 (3)	0.4122 (11)	0.0846 (2)	0.0820 (15)	
H7	0.2537	0.5231	0.0570	0.098*	
C8	0.3286 (3)	0.2242 (12)	0.0670 (2)	0.0863 (16)	
H8	0.3370	0.2078	0.0273	0.104*	
C9	0.3667 (3)	0.0579 (10)	0.10703 (19)	0.0683 (12)	
H9	0.4000	−0.0703	0.0940	0.082*	

### Atomic displacement parameters (Å^2^)

**Table:**

	*U*^11^	*U*^22^	*U*^33^	*U*^12^	*U*^13^	*U*^23^
Sn1	0.0453 (2)	0.0360 (2)	0.0496 (2)	0.000	−0.00011 (16)	0.000
Cl1	0.1927 (17)	0.1008 (12)	0.0620 (8)	0.0479 (12)	0.0451 (10)	0.0020 (8)
F1	0.0875 (19)	0.084 (2)	0.0804 (18)	0.0152 (16)	0.0270 (15)	0.0010 (15)
O1	0.0685 (18)	0.0384 (16)	0.0496 (15)	0.0031 (13)	0.0147 (13)	0.0003 (12)
O2	0.078 (2)	0.0402 (17)	0.0635 (17)	0.0078 (14)	0.0121 (15)	0.0013 (13)
C1	0.065 (3)	0.046 (3)	0.052 (2)	0.003 (2)	0.010 (2)	0.001 (2)
C2	0.171 (6)	0.058 (3)	0.071 (3)	0.017 (4)	0.057 (4)	0.005 (3)
C3	0.049 (2)	0.039 (2)	0.068 (3)	−0.0047 (19)	−0.0033 (19)	−0.004 (2)
C4	0.043 (2)	0.043 (2)	0.053 (2)	−0.0078 (19)	−0.0053 (17)	−0.0040 (19)
C5	0.047 (2)	0.057 (3)	0.057 (3)	−0.003 (2)	0.003 (2)	−0.003 (2)
C6	0.058 (3)	0.062 (3)	0.089 (4)	0.014 (2)	−0.008 (2)	−0.002 (3)
C7	0.085 (4)	0.072 (4)	0.084 (4)	0.003 (3)	−0.032 (3)	0.012 (3)
C8	0.105 (4)	0.098 (4)	0.052 (3)	0.002 (4)	−0.022 (3)	−0.003 (3)
C9	0.071 (3)	0.072 (3)	0.060 (3)	0.008 (3)	−0.008 (2)	−0.016 (2)

### Geometric parameters (Å, °)

**Table:**

Sn1—O1^i^	2.109 (2)	C3—C4	1.492 (5)
Sn1—O1	2.109 (2)	C3—H3A	0.9700
Sn1—C3	2.121 (4)	C3—H3B	0.9700
Sn1—C3^i^	2.121 (4)	C4—C5	1.375 (5)
Sn1—O2^i^	2.537 (3)	C4—C9	1.396 (6)
Sn1—O2	2.537 (3)	C5—C6	1.359 (6)
Cl1—C2	1.728 (5)	C6—C7	1.380 (7)
F1—C5	1.365 (4)	C6—H6	0.9300
O1—C1	1.292 (4)	C7—C8	1.361 (7)
O2—C1	1.207 (4)	C7—H7	0.9300
C1—C2	1.503 (6)	C8—C9	1.377 (7)
C2—H2A	0.9700	C8—H8	0.9300
C2—H2B	0.9700	C9—H9	0.9300
			
O1^i^—Sn1—O1	79.50 (13)	H2A—C2—H2B	107.7
O1^i^—Sn1—C3	110.21 (13)	C4—C3—Sn1	113.7 (3)
O1—Sn1—C3	105.08 (12)	C4—C3—H3A	108.8
O1^i^—Sn1—C3^i^	105.08 (12)	Sn1—C3—H3A	108.8
O1—Sn1—C3^i^	110.21 (13)	C4—C3—H3B	108.8
C3—Sn1—C3^i^	133.6 (2)	Sn1—C3—H3B	108.8
O1^i^—Sn1—O2^i^	54.99 (9)	H3A—C3—H3B	107.7
O1—Sn1—O2^i^	134.28 (9)	C5—C4—C9	115.7 (4)
C3—Sn1—O2^i^	88.52 (13)	C5—C4—C3	121.8 (3)
C3^i^—Sn1—O2^i^	87.82 (13)	C9—C4—C3	122.5 (4)
O1^i^—Sn1—O2	134.28 (9)	C6—C5—F1	118.2 (4)
O1—Sn1—O2	54.99 (9)	C6—C5—C4	124.7 (4)
C3—Sn1—O2	87.82 (13)	F1—C5—C4	117.0 (4)
C3^i^—Sn1—O2	88.52 (13)	C5—C6—C7	118.0 (4)
O2^i^—Sn1—O2	170.72 (12)	C5—C6—H6	121.0
C1—O1—Sn1	100.8 (2)	C7—C6—H6	121.0
C1—O2—Sn1	82.9 (2)	C8—C7—C6	119.8 (5)
O2—C1—O1	121.3 (4)	C8—C7—H7	120.1
O2—C1—C2	124.2 (4)	C6—C7—H7	120.1
O1—C1—C2	114.5 (4)	C7—C8—C9	121.1 (5)
C1—C2—Cl1	113.9 (3)	C7—C8—H8	119.5
C1—C2—H2A	108.8	C9—C8—H8	119.5
Cl1—C2—H2A	108.8	C8—C9—C4	120.6 (4)
C1—C2—H2B	108.8	C8—C9—H9	119.7
Cl1—C2—H2B	108.8	C4—C9—H9	119.7

Symmetry codes: (i) −*x*+1, *y*, −*z*+1/2.
